# Virus-Induced Chaperone-Enriched (VICE) Domains Function as Nuclear Protein Quality Control Centers during HSV-1 Infection

**DOI:** 10.1371/journal.ppat.1000619

**Published:** 2009-10-09

**Authors:** Christine M. Livingston, Marius F. Ifrim, Ann E. Cowan, Sandra K. Weller

**Affiliations:** Department of Molecular, Microbial and Structural Biology, University of Connecticut Health Center, Farmington, Connecticut, United States of America; Oregon Health and Science University, United States of America

## Abstract

Virus-Induced Chaperone-Enriched (VICE) domains form adjacent to nuclear viral replication compartments (RC) during the early stages of HSV-1 infection. Between 2 and 3 hours post infection at a MOI of 10, host protein quality control machinery such as molecular chaperones (e.g. Hsc70), the 20S proteasome and ubiquitin are reorganized from a diffuse nuclear distribution pattern to sequestration in VICE domains. The observation that VICE domains contain putative misfolded proteins suggests that they may be similar to nuclear inclusion bodies that form under conditions in which the protein quality control machinery is overwhelmed by the presence of misfolded proteins. The detection of Hsc70 in VICE domains, but not in nuclear inclusion bodies, indicates that Hsc70 is specifically reorganized by HSV-1 infection. We hypothesize that HSV-1 infection induces the formation of nuclear protein quality control centers to remodel or degrade aberrant nuclear proteins that would otherwise interfere with productive infection. Detection of proteolytic activity in VICE domains suggests that substrates may be degraded by the 20S proteasome in VICE domains. FRAP analysis reveals that GFP-Hsc70 is dynamically associated with VICE domains, suggesting a role for Hsc70 in scanning the infected nucleus for misfolded proteins. During 42°C heat shock, Hsc70 is redistributed from VICE domains into RC perhaps to remodel viral replication and regulatory proteins that have become insoluble in these compartments. The experiments presented in this paper suggest that VICE domains are nuclear protein quality control centers that are modified by HSV-1 to promote productive infection.

## Author Summary

Protein quality control is a protective cellular mechanism by which damaged proteins are refolded or degraded so that they cannot interfere with essential cellular processes. In the event that protein quality control machinery cannot refold or degrade damaged proteins, sequestration of misfolded protein is an alternative protective mechanism for reducing the toxic effects of misfolded protein. Several neurological diseases result from the accumulation of toxic misfolded proteins that cannot be efficiently refolded or degraded. In neurons from patients afflicted with Huntington's disease, misfolded huntingtin protein is sequestered in large aggregates in the nucleus called inclusion bodies. Inclusion bodies also contain protein quality control machinery including molecular chaperones, the proteasome and ubiquitin. Here we report that analogous structures called Virus-Induced Chaperone-Enriched (VICE) domains form in the nucleus of cells infected with Herpes Simplex Virus type 1 (HSV-1). VICE domains contain misfolded protein, chaperones and protein degradation activity. VICE domain formation is efficient in infected cells taxed with high levels of viral protein production. We hypothesize that misfolded proteins that arise in HSV-1-infected cells are sequestered in VICE domains to promote remodeling of misfolded proteins.

## Introduction

Protein quality control (PQC) is essential for maintaining active and properly folded proteins and for degrading aberrantly folded proteins that would otherwise interfere with vital cellular processes. PQC systems consist of a balance between protein refolding machinery (molecular chaperones) and protein degradation machinery (the 26S proteasomal system, ubiquitin conjugation and deconjugation systems and proteasome-independent degradation systems) (reviewed in [1]). PQC systems have been characterized in the cytosol where proteins are produced and initially folded; however, the existence and importance of nuclear PQC has now been recognized. PQC has been implicated in neurodegenerative diseases such as Huntington's and spinal cerebellar ataxia. In diseased cells, misfolded proteins such as mutant huntingtin, mutant ataxin-1 and other abnormal or over-expressed proteins can be detected in nuclear inclusion bodies that contain molecular chaperones, the 20S proteasome, ubiquitin and sometimes PML [2],[3],[4],[5],[6],[7],[8],[9],[10]. Some reports suggest that the formation of nuclear inclusion bodies is cytoprotective, preventing the induction of apoptosis [11],[12]. Additional evidence for nuclear PQC includes the detection of proteolytic activity in nuclear foci under normal cell growth conditions suggesting that turnover of nuclear substrates takes place in specific areas of the nucleus [13]. Unlike cytosolic PQC which utilizes several pathways such as proteolytic degradation/chaperone machinery, lysosomal digestion and autophagy, nuclear PQC appears to rely solely on the ubiquitin-proteasome and chaperone machinery for remodeling and clearance of abnormal proteins [14]. Nuclear PQC thus may be a homeostatic mechanism that prevents misfolded proteins from interfering with nuclear processes [11],[12].

Herpes Simplex Virus type 1 (HSV-1) gene expression occurs in three stages beginning with immediate-early proteins, followed by early replication proteins and finally late structural proteins [15]. In cells infected with Herpes Simplex Virus type 1 (HSV-1), viral gene expression, DNA replication and encapsidation occur in large globular structures designated as replication compartments (RC) in the nucleus [16],[17]. We have recently reported that cellular chaperone proteins such as Hsc70 are reorganized into Virus-Induced Chaperone-Enriched (VICE) domains that form adjacent to nuclear viral RC [18],[19],[20],[21],[22]. Hsc70 is an ATP-dependent constitutively-expressed member of the Hsp70 chaperone family (reviewed in [23],[24],[25]. Hsc70 functions in critical PQC pathways such as binding of nascent polypeptides, identification of misfolded proteins, targeting of misfolded proteins for proteolytic degradation and modulation of protein oligomeric states [26]. In addition to cellular chaperone proteins, VICE domains contain ubiquitinated proteins, the 20S proteasome, and a subpopulation of the viral portal protein UL6 that may be misfolded [18]. Many nuclear HSV-1 processes involve the assembly of multimeric protein complexes such as assembly of viral protein scaffolds (prereplicative sites) that provide platforms for RC formation, formation of the heterotrimeric helicase/primase complex, formation of the dodecameric capsid portal ring and assembly of the viral capsid. Such processes may require the assistance of nuclear molecular chaperones for proper folding.

It is well established that viruses depend on host molecular chaperones for many aspects of their life cycles (reviewed in [27]). For instance bacteriophage λ requires the activities of *E. coli* DnaK (Hsc70 homologue) and DnaJ (DnaK cochaperone) for the release of λP protein from the preprimosomal complex at the origin to initiate phage λ DNA replication [28]. The SV40 large T antigen binds Hsc70 via its J domain perhaps to facilitate the initiation of SV40 DNA replication [29]. Cellular chaperone proteins appear to be important for the life cycle of herpesviruses as well. For example, the Hsp90 inhibitor geldanamycin inhibits the replication of many viruses including HSV-1 [19],[30] and VZV [31]. These studies were extended by the demonstration that Hsp90 activity is required for the nuclear localization of HSV-1 pol (UL30) [19]. Hsc70 localizes to VICE domains adjacent to viral prereplicative sites suggesting that Hsc70 may play a role in RC formation [21]. Hsc70 ATPase activity also appears to be important for RC formation and for the recruitment of RNA pol II to RC [20]. We propose that HSV-1 has evolved to take advantage of the nuclear PQC machinery not only to reduce the toxic effects of misfolded proteins but also to provide access to protein remodeling machinery as needed for protein folding and assembly of multimeric protein complexes.

## Results

### VICE domain formation occurs early during HSV-1 infection and correlates with high levels of viral gene expression

**Figure 1:**
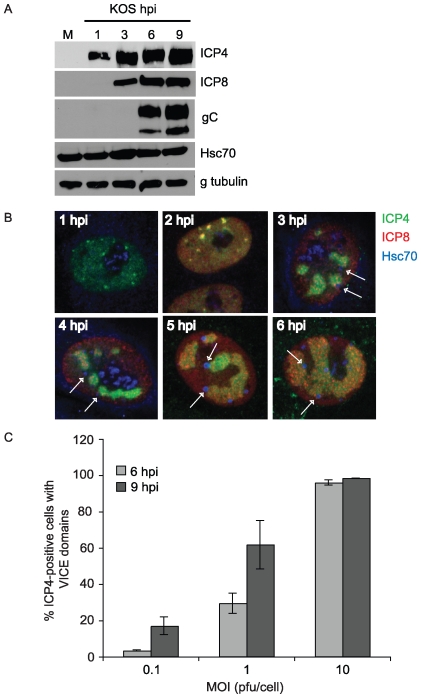
VICE domain formation increases with MOI and duration of infection. In panel A, Vero cells were mock-infected or infected with KOS at an MOI of 10 for 1, 3, 6 and 9 hours. Samples were harvested in 2× DTT-containing SDS sample buffer and resolved on a 10% SDS-PAGE gel. PVDF membranes were reacted with mouse-anti-ICP4, mouse-anti-ICP8, mouse-anti-glycoprotein C (gC), rat-anti-Hsc70 and mouse-anti-γ tubulin antibodies. In panel B, Vero cells were infected with KOS at an MOI of 10 for 1, 2, 3, 4, 5 and 6 hours followed by fixation and permeabilization in preparation for IF analysis. Samples were triple-labeled with mouse-anti-ICP4, rabbit-anti-ICP8 367 and rat-anti-Hsc70 antibodies. Arrows point to VICE domains that formed adjacent to developing RCs. Images were obtained at 63× magnification with 2× digital zoom. In panel C, Vero cells adhered to glass coverslips were infected with KOS at MOIs 0.1, 1, and 10 for 6 and 9 hours. Cells were fixed, permeabilized and double-labeled with mouse-anti-ICP4 and rat-anti-Hsc70 antibodies. At least 100 ICP4-positive cells of each sample were assessed for whether or not they contained VICE domains (Hsc70 foci). ICP4-negative cells never contained Hsc70 foci. The data represent at least 3 independent experiments and error bars were calculated from standard error of the mean.

The induction of molecular chaperone machinery can be triggered by heat shock and other forms of stress that overwhelm the ability of the cell to translate, fold and maintain the integrity of cellular proteins (reviewed in [32]). These homeostatic mechanisms may also be needed in virus-infected cells to handle the increased levels of protein synthesis and the assembly of multimeric protein complexes. In this paper we address the hypothesis that VICE domains represent sites of protein remodeling or quality control. First we address the relationship between viral gene expression and the timing of VICE domain formation. We previously reported that VICE domains are detected adjacent to prereplicative sites in cells in which RC formation has been inhibited by drugs or infection with mutant viruses [21]. In Figure 1, the kinetics of viral gene expression and VICE domain formation were examined in the context of a productive viral infection under conditions in which RC can form. Vero cells were infected for various times at an MOI of 10, and cell lysates were assayed for expression of immediate-early (ICP4), early (ICP8) and late (gC) viral proteins and fixed cells were examined for RC formation (Figure 1A and 1B). At 1 hour-post-infection (hpi), the immediate-early protein ICP4 is detected in cell lysates by Western blot and in nuclear foci by confocal microscopy. We previously reported that in mock-infected cells, Hsc70 is observed in a diffuse nuclear/cytoplasmic pattern with some localization in nucleoli [18],[19], and Figure 1B shows that at 1 hpi Hsc70 is still localized in this pattern. By 2 hpi, low levels of an early protein, ICP8, are detectable in a nuclear diffuse pattern with some localization to developing RCs that contain ICP4. Hsc70 is still localized in a diffuse staining pattern at 2 hpi. At 3 hpi, Hsc70 can be detected in VICE domains adjacent to RC. At this time, levels of ICP4 have increased, and ICP8 is detected by Western blot, indicating that immediate-early and early proteins are expressed. At 4, 5 and 6 hpi, RCs enlarge; however, VICE domains do not appear to change significantly in size or morphology once they have formed. The true late protein gC is detected at 6 hpi indicating that viral DNA synthesis has taken place. As we have previously reported, the protein levels of Hsc70 do not appear to change significantly during the first 18 hours of HSV-1 infection (Figure 1A) [18]. These experiments demonstrate that VICE domain formation occurs early during HSV-1 infection and correlates with early gene expression. This result is consistent with previous observations that immediate-early proteins that are required for efficient early gene expression are also required for efficient VICE domain formation [18],[20].

### VICE domain formation is efficient at high MOI

Infection at high MOI is expected to result in higher levels of HSV-1 gene expression per cell than infection at low MOI. In order to test the hypothesis that VICE domain formation correlates with gene expression, we examined the relationship between MOI and the efficiency of VICE domain formation. Vero cells were infected with KOS at MOIs ranging from relatively low (0.1 pfu/cell) to relatively high (10 pfu/cell). Using ICP4 as a marker for viral infection and Hsc70 as a marker for VICE domains, we determined the percentage of infected cells that contained VICE domains. Figure 1C shows that only about 3% of the cells that were infected at an MOI of 0.1 for 6 hours contained VICE domains as compared to 30% at an MOI of 1 and about 96% at a high MOI of 10. More efficient VICE domain formation at high MOI may be due to increased levels of viral protein synthesis.

Next we asked if the defect seen at low MOI reflects an inability to form VICE domains or a delay in their formation. When cells were harvested at 9 hpi, we observed that 17% of cells infected at an MOI of 0.1 contained VICE domains. At an MOI of 1, the percentage of infected cells that contain VICE domains at 9 hpi increased to 62%. Thus it appears that VICE domain formation is delayed at low MOI compared to high MOI perhaps reflecting a slower establishment of gene expression.

**Figure 2:**
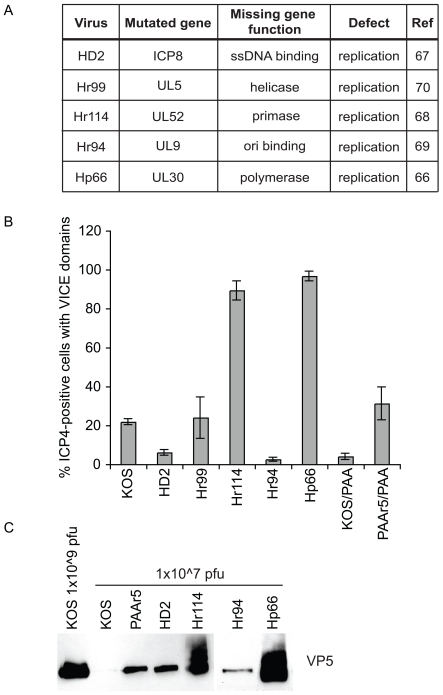
Viral stocks with high particle-to-pfu ratio induce the formation of VICE domains. The replication mutant viruses used in this study are detailed in panel A. In panel B, Vero cells were infected with KOS, HD2 (ICP8 mutant), Hr99 (UL5 mutant), Hr114 (UL52 mutant), Hr94 (UL9 mutant), Hp66 (UL30 mutant) or PAAr5 (PAA-resistant KOS) for 6 hours at an MOI of 2 in the presence or absence of 400 ug/mL PAA (where indicated). At least 100 ICP4-positive cells of each sample were assessed for whether or not they had formed VICE domains. The data represent 3 independent experiments and error bars were calculated from standard error of the mean. In panel C, Viral stocks were normalized to 1e7 pfu/mL based on known titers and loaded directly to an 8% SDS-PAGE gel. Proteins were transferred to a PVDF membrane and blotted for the major capsid protein VP5.

We showed above that VICE domain formation correlated with the expression of viral early genes. Next we asked whether VICE domain formation requires the presence of particular early proteins that are essential for HSV-1 DNA replication. Vero cells were infected with viral DNA replication mutants (Figure 2A) at an MOI of 2. Figure 2B shows that infection with two viral mutants, Hr114 or Hp66, resulted in an extremely high efficiency of VICE domain formation where approximately 90–100% of the ICP4-positive cells contained VICE domains. On the other hand, at this MOI, 20% or less of the cells infected with KOS, HD2, Hr99 and Hr94 exhibited VICE domains. Thus two DNA replication-negative mutants exhibit higher than expected efficiency of VICE domain formation, and three other DNA replication-negative mutants exhibit reduced VICE domain formation. Thus it appears that the behavior of Hr114 and Hp66 is not related to their defect in viral DNA synthesis. To further explore the relationship between VICE domain formation and the ability to synthesize viral DNA, we tested KOS-infected cells in the presence of the viral DNA synthesis inhibitor phosphonoacetic acid (PAA). This treatment reduced the percentage of infected cells containing VICE domains (Figure 2B). To ensure that impaired VICE domain formation was not due to the affects of PAA, Vero cells were infected with a PAA-resistant mutant PAAr5 (obtained from Dr. Donald Coen) in the presence of PAA. Under these conditions, VICE domain formation was restored (Figure 2B). The PAA experiments indicate that efficient VICE domain formation generally requires viral DNA synthesis. On the other hand the behavior of Hr114 and Hp66 indicates that some mutant stocks can induce VICE domain formation even though they fail to synthesize viral DNA.

To explain this discrepancy we considered the possibility that stocks of Hr114 and Hp66 contain a high number of defective particles resulting in a higher than normal particle-to-PFU ratio. In this scenario, infection of cells at an MOI of 2 based on the infectious viral titer would not reflect the presence of noninfectious particles that may also still stimulate gene expression. In order to estimate the number of viral particles in mutant and wild type stocks, 1×10^7^ plaque forming units (PFU) were concentrated, subjected to SDS-PAGE and immunoblotted with antibody against the major capsid protein VP5 to detect viral capsids (Figure 2C). Interestingly, the Hr114 and Hp66 viral stocks displayed increased levels of VP5 compared to KOS and the other mutant viruses. In fact VP5 was barely detectable in the KOS stock. The other viral mutants exhibited intermediate particle-to-pfu ratios. For comparison, undiluted KOS (1×10^9^ pfu) was also included to ensure that VP5 can be detected in a concentrated KOS stock. The stocks that resulted in the highest proportion of infected cells with VICE domains also had the highest particle-to-pfu ratios. Infection with stocks that contain an intermediate particle-to-pfu ratio, HD2 and Hr94, resulted in inefficient VICE domain formation that is comparable to KOS infection in the presence of PAA (compared with KOS infection). This may reflect their defect in DNA replication despite the fact that these stocks have a higher particle-to-PFU ratio than KOS. This is consistent with our previous observations that DNA synthesis is required for efficient VICE domain formation [18]. The efficient VICE domain formation in cells infected with Hr114 and Hp66 may reflect gene expression levels higher than anticipated based on the infectious titer. Thus it is possible that abnormally high levels of protein synthesis occur in cells infected with these stocks despite the fact that DNA synthesis is inhibited. In cells infected with KOS at a relatively low MOI, VICE domain formation may require the robust levels of protein synthesis that generally occur following viral DNA synthesis. These results are consistent with the results shown in Figure 1 demonstrating that VICE domain formation occurs more efficiently at higher MOIs.

### VICE domains contain misfolded protein

**Figure 3:**
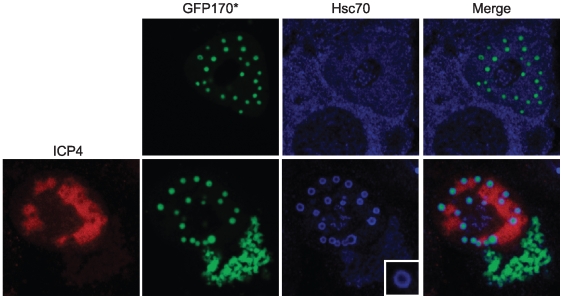
A model misfolded protein is localized to VICE domains. Vero cells were transfected with 1 ug of plasmid DNA encoding the model misfolded protein GFP170* [2]. Following about 16 hours of protein expression, the cells were infected with KOS at an MOI of 10 for 6 hours. Cells were fixed, permeabilized and labeled with rabbit-anti-ICP8 367 and rat-anti-Hsc70 antibodies. The inset in the bottom row shows an enlarged image of a VICE domain containing GFP170*.

Fu *et al* have reported that nuclear inclusions in normal cells contain ubiquitin, the 20S proteosome, Hsp70 and misfolded proteins [2]. Our observation that HSV-1 infection causes redistribution of Hsc70, the 20S proteosome and ubiquitin into VICE domains led us to speculate that one role of VICE domain formation is to process or refold proteins. The observation that a subpopulation of UL6, the HSV-1 portal protein, is found in VICE domains is consistent with this hypothesis as UL6 is assembled into a dodecameric ring structure during capsid formation. It is possible that improperly folded monomers or subcomplexes of UL6 are localized to VICE domains. To test the hypothesis that VICE domains contain misfolded protein, we obtained a model misfolded protein (GFP170*) that has been shown to form nuclear and cytoplasmic inclusions [2]. Vero cells transiently expressing GFP170* were infected with KOS 14 hours following transfection. Cells were fixed at 6 hpi and labeled for ICP4 (an RC marker) and Hsc70 (a VICE domain marker). The bottom row of Figure 3 shows that GFP170* was detected in VICE domains adjacent to RCs consistent with the notion that VICE domains contain misfolded proteins. Interestingly Hsc70 appears to form a shell around GFP170* in VICE domains. Although we often observe Hsc70 in a ring-like pattern in VICE domains under normal infection conditions, this pattern seems to be exaggerated when misfolded protein is over-expressed by transfection. These observations are consistent with previous reports that chaperones are able to form a shell around aggregated misfolded proteins [33],[34].

### Hsc70 is specifically recruited to VICE domains and not to nuclear inclusions in uninfected cells

We have previously reported that both Hsp70 and Hsc70 are found in VICE domains [18],[19]. On the other hand, Fu *et al* showed that although Hsp70 was observed in nuclear inclusions that contain misfolded proteins, these nuclear inclusions did not appear to contain Hsc70 [35]. Consistent with these results, the top row of Figure 3 shows that Hsc70 does not localize to nuclear inclusions in the absence of infection. In summary, we have shown that VICE domains and nuclear inclusions both contain model misfolded protein; however, Hsc70 is specifically found in VICE domains and not in nuclear inclusions in the absence of infection. We cannot exclude the possibility that Hsc70 may be recruited to nuclear inclusions that contain other types of misfolded proteins; however, from the results shown we suggest that HSV-1 may specifically require Hsc70 activity in addition to Hsp70.

### VICE domains are resistant to detergent treatment

**Figure 4:**
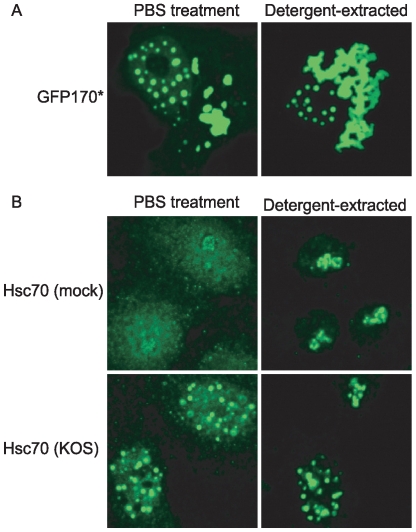
VICE domains are resistant to detergent extraction. (A) Vero cells were transfected with 1 ug GFP170* [2]. Following 16 hours of protein expression, cells were treated with PBS (left panel) or 0.5% TX-100 detergent extraction buffer (right panel) for 2 minutes on ice. Cells were then fixed in 4% paraformaldehyde, permeabilized in 1% TX-100 and analyzed for localization of GFP170*. (B) Vero cells were mock infected or infected with KOS at an MOI of 10. Following 6 hours of infection, cells were treated with PBS or detergent extraction buffer as described in (A). Hsc70 localization was detected with a rat-anti-Hsc70 (Stressgen) antibody. Imaging was performed on a Zeiss LSM 410 confocal microscope. The gain was set to visualize the diffuse Hsc70, resulting in saturation of the VICE domains.

Nucleosolic and cytosolic proteins can be extracted from cells with a TX-100 detergent buffer leaving behind proteins that are bound to matrix (e.g. cytoskeleton, nuclear matrix, chromatin) or insoluble proteins. A defining feature of nuclear inclusions is that they are resistant to detergent extraction, probably because they contain insoluble misfolded proteins [36]. Figure 4A shows that in cells transfected with GFP170*, nuclear inclusions were resistant to detergent treatment. Next we observed that Hsc70 is extractable from the nucleus and cytoplasm of uninfected cells; whereas, Hsc70 localized to nucleoli was resistant to detergent treatment (compare Figure 4B top left and right panels). This result indicates that the detergent extraction buffer was effective in removing a subpopulation of Hsc70 from cells. We surmised that if VICE domains share properties with nuclear inclusions, they would be resistant to detergent treatment. Figure 4B bottom panels left and right show that Hsc70 is detected in VICE domains even after detergent extraction of nucleosolic and cytosolic proteins.

### Protease activity is detected in VICE domains

We previously reported that the 20S subunit of the 26S proteasome is localized to VICE domains during productive infection [18]. Although proteolysis was previously thought to occur only in the cytoplasm, it is now recognized that centers of proteolysis exist in the nucleus (reviewed in [37]). We next asked whether the 20S proteasome is catalytically active within VICE domains. Nuclei of infected or uninfected HFF-1 cells were microinjected with a fluorescent substrate, DQ-Ovalbumin, which is labeled with a fluorescent BODIPY FL dye that exhibits bright fluorescence in the green channel upon proteolytic degradation (Molecular Probes). DQ-OVA is conjugated with quenching dyes such that only 3% of the green fluorescent dye is detected when the substrate is intact [13]. Upon cleavage by the 20S proteasome, quenching is relieved and fluorescently labeled peptide can be detected.

**Figure 5:**
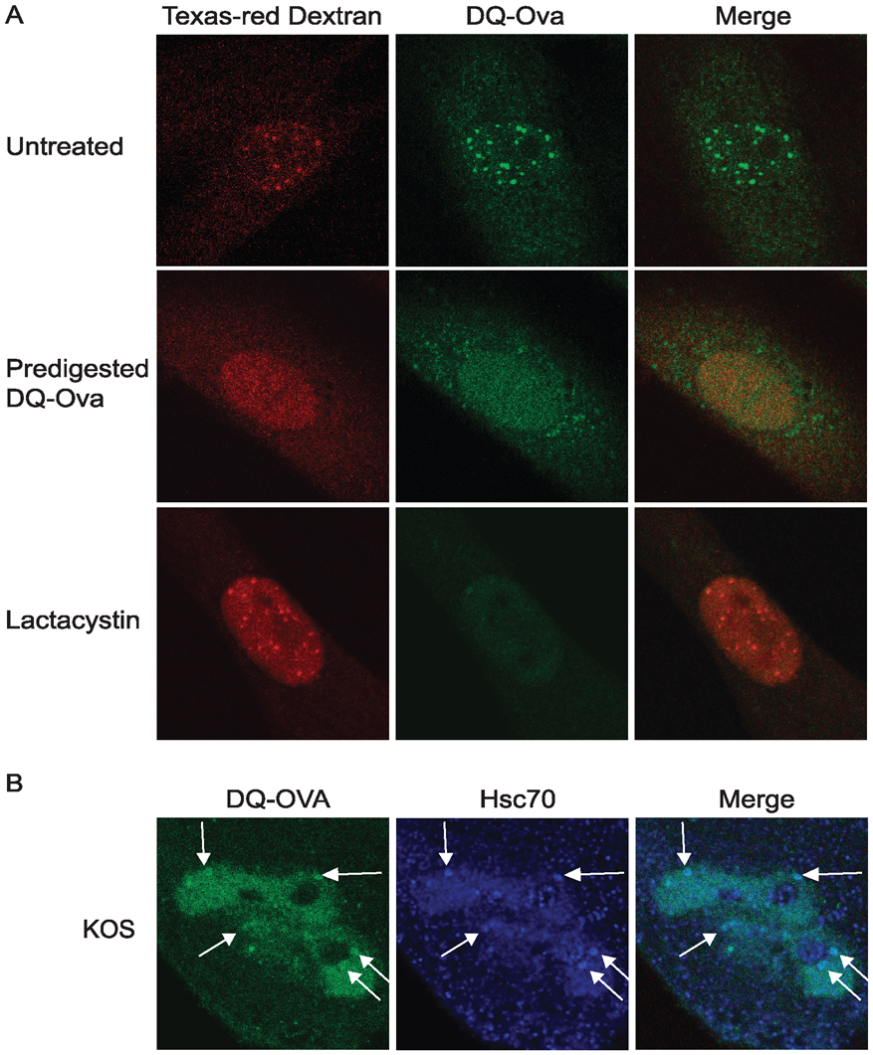
Protease activity is detected in VICE domains. (A) HFF-1 cells were plated in dishes containing embedded glass coverslips and were co-microinjected with TX red dextran and the 20S proteasomal substrate DQ-OVA. Images of live cells are shown. In the middle row, cells were co-microinjected with TX red dextran and DQ-OVA that was predigested in the presence of trypsin. In the bottom row, cells were co-microinjected with TX red dextran, DQ-OVA and lactacystin. (B) HFF-1 cells plated in dishes with embedded coverslips were infected with 2 pfu/cell KOS and microinjected 5 hours later with TX red dextran and DQ-OVA. Following about 20–30 min of proteolytic cleavage, the cells were fixed, permeabilized and stained for Hsc70.

In the experiment shown in Figure 5A, uninfected HFF-1 cells were co-microinjected with DQ-OVA and the injection control TX red dextran which cannot diffuse across the nuclear membrane. As previously reported, nuclear foci of proteolytic activity could be detected in uninfected cells [13]. Foci of proteolytic activity were not detected in cells microinjected with Ova predigested with trypsin (Figure 5A, middle row) or in cells co-microinjected with the proteasome inhibitor lactacystin (Figure 5A, bottom row). These controls indicate that the DQ-OVA substrate is specifically degraded by the 20S proteasome in nuclear foci in uninfected cells.

Next, we asked whether proteolytic activity can be detected in VICE domains. HFF-1 cells were infected at an MOI of 2 and nuclei were microinjected with DQ-OVA and TX red dextran (data not shown) at 5–6 hpi. At 20–30 minutes post injection cells were fixed, permeabilized and treated with antibodies against Hsc70. In Figure 5B cleavage of DQ-Ova was detected in structures that resemble RCs and in foci that colocalize with Hsc70. These data show that proteolytic activity is detected in VICE domains.

### Homotypic ubiquitin chains are not detected in VICE domains

Polyubiquitin chains are formed by the linkage of each ubiquitin monomer to one of 7 lysine residues (K6, K11, K27, K29, K33, K48 or K63) of another ubiquitin monomer. The best-characterized polyubiquitin chains are those that utilize lysines 48 and 63. Specific lysine linkages are one determinant in the fate of the target protein [38]. Proteins conjugated with K48 ubiquitin chains are generally targeted for proteasomal degradation; whereas, K63 chains generally signal a non-proteolytic fate such as sequestration in inclusion bodies [39],[40]. Monoclonal antibodies (FK1 and FK2) were previously used to detect polyubiquitinated proteins in VICE domains [18]. Determination of the lysine linkages used in the polyubiquitin chains found in VICE domains may reveal whether or not substrates in VICE domains are destined for proteolytic degradation. We obtained expression plasmids carrying either HA-tagged wt ubiquitin or mutant HA tagged ubiquitin that can only polymerize using lysine 48 (K48 ubiquitin) or lysine 63 (K63 ubiquitin). The other 6 lysines of each of the mutant ubiquitin constructs were mutated to arginine, and thus cannot be used for polymerization. Wild type ubiquitin, on the other hand, can utilize any of the seven lysines for polymerization.

**Figure 6:**
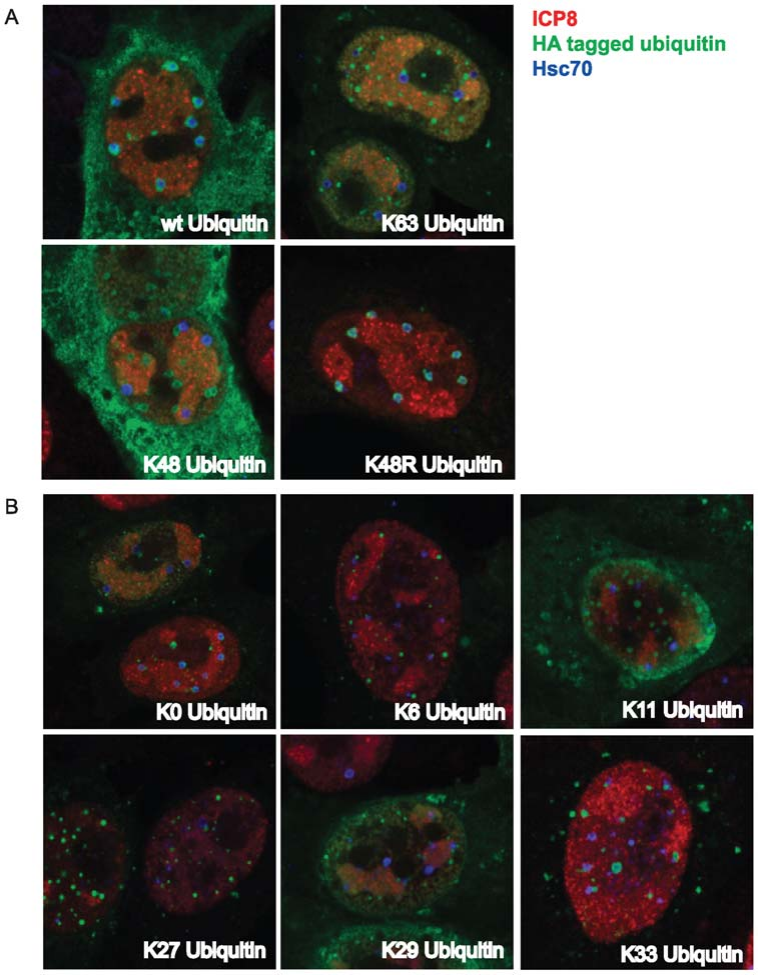
Homotypic ubiquitin chains are not detected in VICE domains. Vero cells adhered to glass coverslips were transfected with 1 ug of pRK-HA-Ub constructs encoding wt, K63, K48 and K48R Ub (panel A) and K0 (monomeric), K6, K11, K27, K29 and K33 Ub (panel B). Following 16–18 hours of protein expression, transfected cells were infected with KOS at an MOI of 10 for 6 hours. At harvest, cells were prepared for IF analysis and labeled with mouse-anti-HA tag, rabbit-anti-ICP8 367 and rat-anti-Hsc70 antibodies. Images were taken at 63× magnification with 2× digital zoom. Merged images of green, red and far-red channels are shown.

In the experiment shown in Figure 6A, Vero cells were transfected with constructs encoding HA-wt ubiquitin, HA-K48 ubiquitin or HA-K63 ubiquitin for 16 hours before superinfection with 10 pfu/cell HSV-1. Infected cells were fixed at 6 hours post infection and labeled for ICP8, HA tag, and Hsc70. Consistent with antibody staining for poly-ubiquitin, we observed that wt ubiquitin colocalized with VICE domains (Figure 6A); however, we were surprised that neither K48 nor K63 ubiquitin chains colocalized with VICE domains (Figure 6A). K63 ubiquitinated proteins were detected in nuclear foci often adjacent to VICE domains raising the possibility that the two foci are distinct but interrelated in function.

We next hypothesized that atypical ubiquitin chains linked via K6, K11, K27, K29 or K33 may be localized to VICE domains. We obtained a construct encoding ubiquitin that can only polymerize using the K33 linkage, and we generated mutants that can only polymerize using K6, K11, K27 or K29 linkages. Cells expressing each construct were infected with KOS at an MOI of 10 for 6 hours. In each case, we did not observe significant colocalization of ubiquitin foci and Hsc70 foci (Figure 6B). Monomeric ubiquitin (all lysines converted to arginine) was also detected in foci distinct from VICE domains. A ubiquitin mutant (K48R) that can polymerize using any of the lysines except for K48 localizes to VICE domains in a subpopulation of infected cells suggesting that at least two lysine linkages are utilized for polymerization (Figure 6A). Mass spectroscopic analysis has revealed that polyubiquitin chains can also form branched structures that utilize combinations of the 7 lysine residues (reviewed in [41]). These data suggest that the wt ubiquitin detected in VICE domains forms branched structures or chains that use more than one lysine linkage.

### Hsc70 is dynamically associated with VICE domains

**Figure 7:**
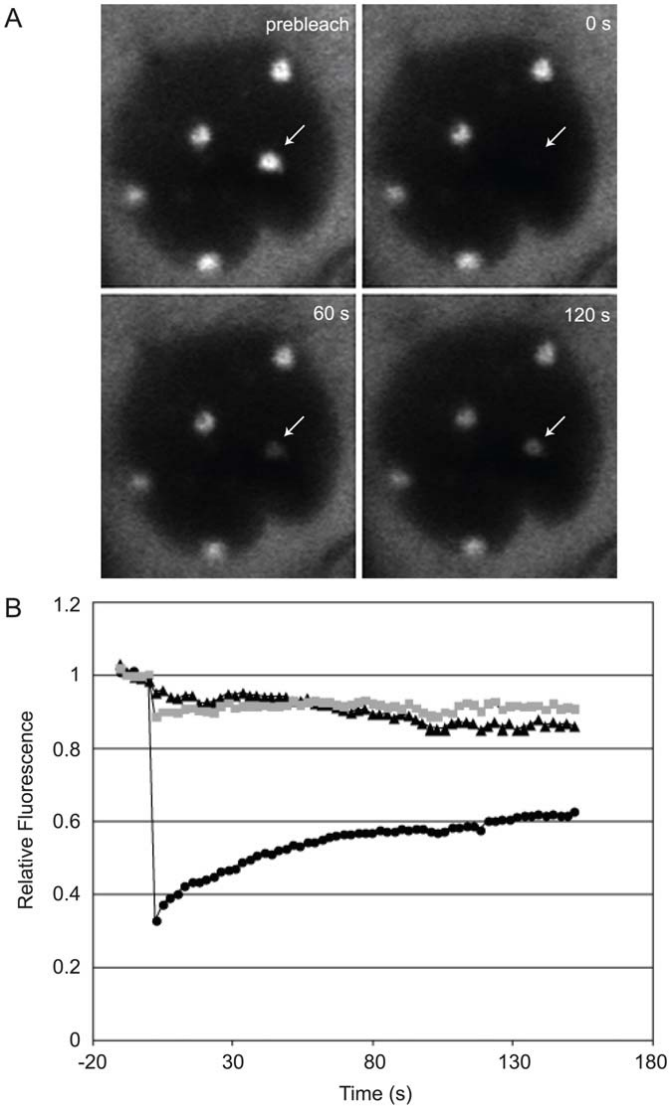
FRAP analysis of Hsc70-GFP in VICE domains. Vero cells adhered to a coverslip dish were transfected with 1 ug of a plasmid encoding wt Hsc70-GFP. Approximately 16 hours post transfection, cells were infected with KOS at an MOI of 10. At 5 hours post infection, live infected cells were imaged on the LSM 510 Meta confocal microscope. At time 0, Hsc70-GFP in one VICE domain was photobleached using 100% argon laser power, and subsequent images collected at ∼2.5 s intervals. (A) Single images from the time series are shown; arrow indicates the VICE domain selected for photobleaching. (B) Fluorescence intensity in the bleached VICE domain (circles), a VICE domain that was not photobleached (squares) and an equivalently sized area of the nucleoplasm (triangles) is shown. Data are normalized to prebleach intensity and corrected for bleaching during monitoring. The experiment shown is representative of at least three independent experiments each involving analysis of numerous infected cells.

FRAP analysis has been used to demonstrate that chaperones and misfolded proteins are dynamically associated with nuclear inclusion bodies [2],[33]. We hypothesize that a freely diffusing population of Hsc70 may shuttle between VICE domains and the nucleoplasm to sample the nuclear environment for misfolded proteins. To test this hypothesis, Vero cells transiently expressing GFP-Hsc70 were infected with HSV-1 at an MOI of 10. Since Hsc70 nuclear foci are not present in uninfected cells, we presumed that cells containing nuclear foci of Hsc70 at 5 hpi were infected. In the experiment shown in Figure 7, one VICE domain per cell was rapidly photobleached, and recovery was assessed over 5 min. GFP-Hsc70 in VICE domains partially recovered (black circles) indicating a rapid exchange between Hsc70 in VICE domains and freely diffusing Hsc70 in the nucleus. The t1/2 for recovery (∼1 min) corresponds to the apparent off rate for dissociation of Hsc70 from VICE domains (koff∼0.01 s-1). Complete recovery was not achieved indicating an immobile fraction (∼50%) of Hsc70 tightly associated with VICE domains. On the other hand, the fluorescence intensity of a neighboring unbleached VICE domain (gray squares) did not appear to change during the experiment. Some loss of fluorescence intensity was observed in an equivalent area of the nucleoplasm (black triangles) possibly due to movement of fluorescent Hsc70 into the bleached VICE domain. We conclude that under normal infection conditions some Hsc70 in VICE domains is in rapid equilibrium with freely diffusing Hsc70 in the surrounding nucleoplasm.

### Heat shock at 42°°°°C causes redistribution of Hsc70 from VICE domains into RC

**Figure 8:**
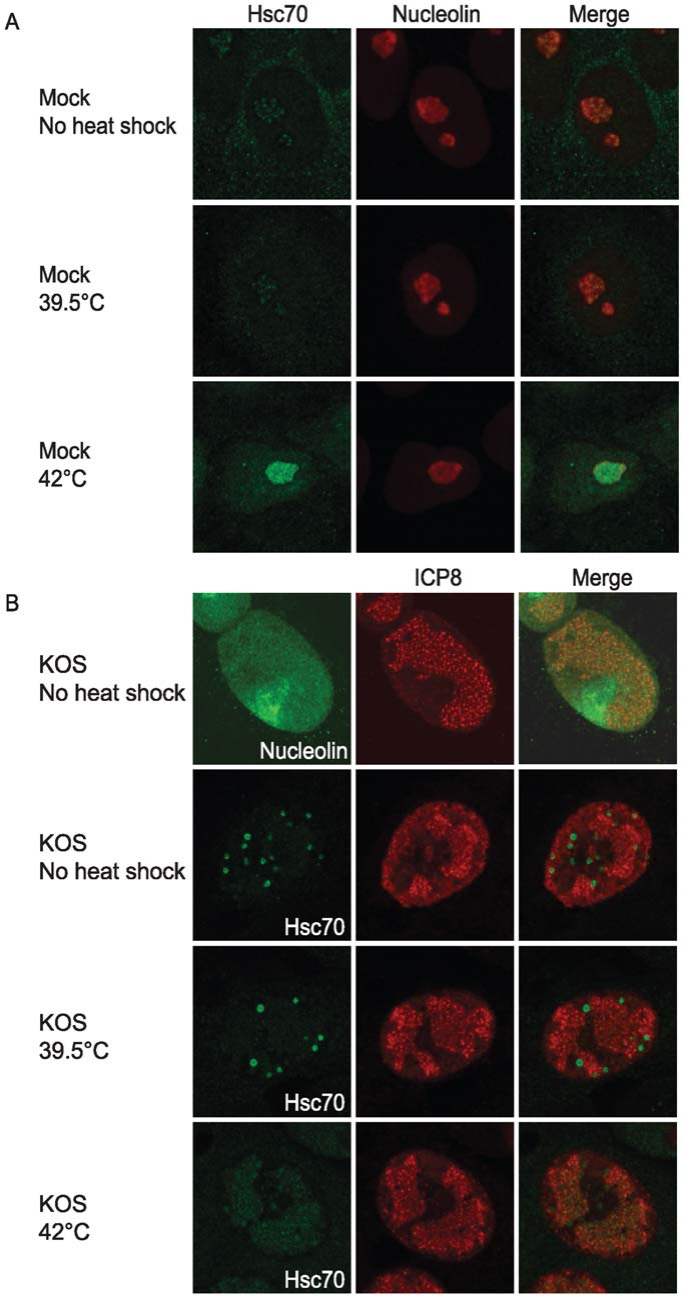
Hsc70 localizes to replication compartments in 42°°°°°°C heat-shocked cells. In panel A, mock infected cells were left at 37°C or heat shocked at 42° or 39.5°C for 1 hour followed by harvest for IF analysis of Hsc70 localization. In panel B, Vero cells were infected with KOS at an MOI of 10. Top row: infected cells were double labeled for nucleolin and ICP8. Bottom three rows: At 5 hours post infection, cells were either left at 37°C or transferred to a 42°C incubator for 1 hour. All samples were harvested at 6 hours post infection and were double labeled for Hsc70 and ICP8.

One aspect of the cellular response to heat stress at 42°C includes the translocation of Hsc70 to the nucleolus [42],[43],[44]. To confirm this observation in the absence of virus, uninfected Vero cells were incubated at 37°C, 39.5°C or 42°C for 1 hour, fixed and prepared for immunofluorescence staining of Hsc70 and nucleolin. In cells incubated at 37°C and 39.5°C, Hsc70 was mostly cytoplasmic and nuclear diffuse (as well as some nucleolar localization) (Figure 8A). In contrast, Hsc70 was detected in or surrounding the nucleoli of 42°C-treated cells. Thus, the translocation of Hsc70 to the nucleoli of cells heat-shocked at 42°C appears to represent a normal cellular response to heat shock.

The relocalization of Hsc70 to VICE domains during infection suggests that HSV-1 may disrupt the normal cellular response to stress by reorganizing cellular protein remodeling machinery such as Hsc70 to VICE domains. We next asked whether Hsc70 in infected cells can translocate to nucleoli during heat shock at 42°C. Previous electron microscopy studies have shown that nucleolar components aggregate and become more compact and electron dense at late times post infection [45],[46]. More recently nucleolin was shown to be dispersed during HSV-1 infection at an MOI of 10 when analyzed at 8 hpi or later [47],[48]. In our hands, at 6 hpi at an MOI of 10, nucleolin is detected in the nucleoli (Figure 8B top row) although the pattern is slightly more diffuse than that seen in uninfected cells suggesting that nucleoli are modified but still intact under these conditions. Vero cells were infected at an MOI of 10 followed by heat shock at 42°C for 1 hour at 5 hpi. All samples were harvested after 1 hour of heat shock for a total infection time of 6 hours. We found that regardless of the temperature treatment, RC (as detected by ICP8 staining) were intact suggesting that drastic changes in nuclear architecture did not take place during heat shock. Cells that were incubated at 37°C during the entirety of infection exhibited Hsc70 in VICE domains adjacent to RC as expected. Surprisingly, VICE domains were not detected in cells heat shocked at 42°C; in fact, Hsc70 was no longer in defined foci but rather appeared to be recruited to RC. VICE domains did not reform when infected cells were analyzed 2 hours after heat shock (data not shown). These data combined with the data presented in Figure 7 confirm that VICE domains are dynamic heat stress-responsive structures and that Hsc70 is recruited into RC during heat stress.

### Viral RC proteins become insoluble at 42°°°°C heat shock

**Figure 9:**
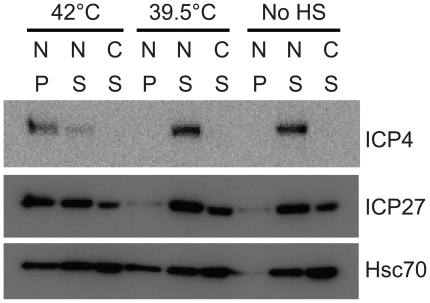
Viral proteins lose solubility during 42°°°°°°C heat shock. Vero cells were infected with 10 pfu/cell KOS for 6 hours. During the 5^th^ hour of infection, samples were either left at 37°C or heat shocked for 1 hour at 42°C or 39.5°C. Whole cell extract was fractionated into nuclear (N) soluble (S), nuclear insoluble (P) and cytosolic (C) fractions as described in Materials and Methods, resolved on 10% SDS-PAGE gels and transferred to PVDF membrane. Membranes were probed with monoclonal antibodies specific for ICP4, ICP27 or Hsc70.

Previous reports have indicated that nuclear proteins are particularly sensitive to heat shock [49]. For example, in a study that compared the stability of firefly luciferase in the nucleus versus cytosol during heat shock at 42°C, nuclear luciferase was more susceptible to thermal denaturation and was slower to recover activity compared to cytosolic luciferase [50]. Furthermore, Hsp70 and Hsc70 have been shown to refold and compartmentalize unfolded nuclear proteins in uninfected heat shocked cells [51]. We hypothesized that Hsc70 is redistributed from VICE domains into RC during heat shock at 42°C in order to remodel proteins that are damaged by the high temperature. To determine if viral RC proteins become insoluble during 42°C heat shock, we fractionated KOS-infected cells that were heat shocked at 39.5°C or 42°C into nuclear (N) soluble (S), nuclear insoluble (P) and cytosolic (C) fractions followed by Western analysis of Hsc70 and two viral proteins that are found in RC, ICP4 and ICP27, respectively. In the experiment shown in Figure 9, ICP4 and ICP27 were detected in the nuclear soluble fraction under normal infection conditions at 37°C. ICP27 shuttles between the nucleus and the cytoplasm and thus was also detected in the cytosolic fraction as was Hsc70. Heat shock of infected cells at an intermediate temperature (39.5°C) did not appear to affect the solubility or distribution of ICP4 and ICP27; however, a portion of Hsc70 was detected in the nuclear insoluble fraction. Heat shock of infected cells at 42°C resulted in the detection of ICP4 and ICP27 in the nuclear insoluble fraction. Consistent with these results, the RC protein ICP8 was soluble under normal infection conditions and following heat shock at 39.5°C but insoluble following heat shock at 42°C (data not shown). We conclude that heat shock of infected cells at 42°C results in the insolubility of nuclear viral RC proteins which may lead to recruitment of Hsc70 into RC in response to the presence of misfolded proteins. Although heat shock treatment would be expected to denature cellular as well as viral proteins, it is of interest that Hsc70 is enriched in replication compartments and not in the nucleolus.

### Heat shock at 42°°°°°C is deleterious to viral productivity

**Figure 10:**
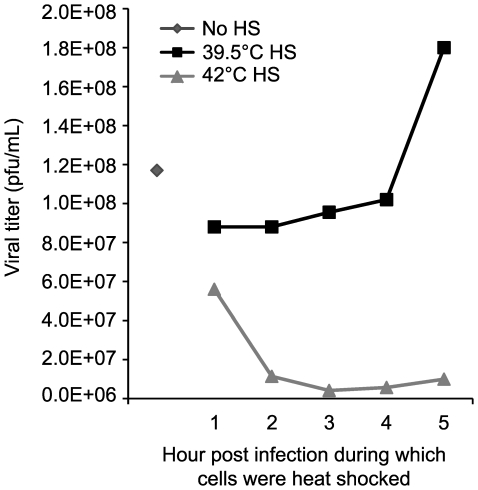
Viral productivity decreases following heat shock. Vero cells were infected with 10 pfu/cell and heat shocked at either 39.5°C or 42°C during the indicated hour post infection before shift back to 37°C. Cell-associated and supernatant virus was harvested 24 hours post infection followed by titration on Vero cell monolayers in triplicate.

To determine the effect of 42°C heat shock on HSV-1 productivity, we performed a heat shock time course experiment and assayed for viral productivity (Figure 10). Infected Vero cells were heat shocked for 1 hour at the indicated hpi and then returned to 37°C; virus was harvested at 24 hpi and titered on Vero cell monolayers. In the experiment shown in Figure 10, heat shock at 42°C resulted in a decrease in viral productivity at each time post infection. Heat shock at 39.5°C did not significantly affect viral productivity compared with infection of non-heat shocked cells which resulted in a titer of 1.2×10^8^ pfu/mL. Interestingly, infected cells that were heat shocked at 42°C as early as 2 hours post infection exhibited serious declines in virus production suggesting that 42°C heat shock irreversibly affects viral productivity.

## Discussion

Several observations are made in this paper concerning the reorganization of the cellular heat shock protein Hsc70 and the formation and function of Virus-Induced Chaperone-Enriched (VICE) domains during HSV-1 infection. The formation of VICE domains correlates with early gene expression and is most efficient at high multiplicities of infection or during infection with virus stocks that contain a high number of particles relative to plaque-forming-units. Thus VICE domain formation appears to correlate with robust viral gene expression. Furthermore, VICE domains are dynamic structures that contain a model misfolded protein, molecular chaperones, active proteolytic machinery and are resistant to detergent extraction. These properties suggest that VICE domains are similar to nuclear inclusions that are formed in cells expressing misfolded proteins such as mutant huntingtin or ataxin-1 protein [2],[3],[4],[5],[6],[7],[8],[9],[10]. In infected cells that have been subjected to heat shock at 42°C, VICE domains are disrupted and Hsc70 is recruited into replication compartments (RC). Under these conditions, several viral proteins become insoluble suggesting that Hsc70 is recruited to RC in response to the presence of damaged proteins. VICE domains thus appear to represent centers of nuclear protein quality control (PQC). We propose that HSV-1 has evolved to take advantage of the nuclear PQC machinery not only to reduce the toxic effects of misfolded proteins but also to provide access to protein remodeling machinery as needed for protein folding and assembly of multimeric protein complexes.

### Possible functions of VICE domains during infection

VICE domain formation may be a mechanism to sequester and process misfolded proteins that arise as a result of robust HSV-1 gene expression. Several studies have demonstrated that sequestration of misfolded proteins into cytosolic or nuclear inclusions reduces their toxicity to the cell, and disruption of inclusion formation induces cell death [11],[12],[52],[53]. VICE domains may represent cytoprotective structures that delay premature apoptosis by sequestering misfolded proteins. Interestingly, HSV-1 infection has been shown to both induce and inhibit components of the apoptotic response pathway (reviewed in [54]).

VICE domains may also serve as storage compartments for chaperone machinery that can be released as needed during HSV-1 infection. The sequestration of Hsc70 in VICE domains provides a dynamic pool of protein remodeling machinery that can be used for protein folding and for the assembly of multimeric viral complexes. For instance, monomeric protein subunits that make up larger structures such as the HSV-1 heterotrimeric helicase/primase complex (UL5/UL8/UL52), the portal complex (UL6 dodecameric ring) or capsids themselves may require the assistance of host molecular chaperones. Indeed we previously reported that individual members of the UL5/8/52 helicase/primase complex aggregated in uninfected cells when expressed without their protein partners [55]. We hypothesize that VICE domains contain a concentrated pool of protein remodeling machinery such as Hsc70 that can be accessed during HSV-1 infection. The dynamic shuttling of Hsc70 between VICE domains and the surrounding nucleoplasm (Figure 7) is consistent with this hypothesis.

During heat stress, the ability to recruit Hsc70 into RC may be especially important given that 42°C heat shock reduces the solubility of viral proteins (Figure 9). Interestingly, it was previously demonstrated that heat stress can inactivate nuclear replication/transcription proteins such as eukaryotic DNA pol α and DNA pol ε, prokaryotic DNA pol III and RNA pol [56] as well as eukaryotic TOPO I protein [57] and that over-expression of Hsc70 can restore activity under these conditions. It is possible that in HSV-1-infected cells, Hsc70 is recruited to RC to reactivate cellular and viral proteins that are critical for viral gene expression and/or DNA replication. Indeed, the Sandri-Goldin laboratory proposed that Hsc70 works in conjunction with the immediate-early protein ICP27 to resolve stalled transcription complexes in RC [20]. Our observation that Hsc70 exchanges rapidly between VICE domains and the surrounding nucleoplasm raises the possibility that Hsc70 shuttles in and out of RC to resolve protein aggregates. In summary, we suggest that HSV-1 has evolved to take advantage of the nuclear PQC machinery not only for the prevention of apoptosis but also to rapidly access this machinery as needed for protein folding and assembly of multimeric protein complexes.

### Hsc70 is recruited to VICE domains and not to GFP170* nuclear inclusions

The observation that Hsp70, but not Hsc70, is associated with GFP170* nuclear inclusions [2] suggests that Hsp70 and Hsc70 play distinct roles in nuclear protein quality control. It appears that Hsc70 is specifically recruited to VICE domains during infection. Although in some assays Hsp70 and Hsc70 play similar roles, it is possible that some proteins are recognized by one or the other specifically. For instance, previous reports have specifically implicated Hsc70 in specific types of protein disaggregation such as the uncoating of clathrin coated endocytic vesicles [58], ribosomes in the nucleoli of heat shocked cells [42] and aggregated luciferase [59]. The observation that Hsc70 is specifically recruited to VICE domains suggests that Hsc70 may perform a specialized function during HSV-1 infection.

### Ubiquitinated substrates in VICE domains may be conjugated to branched poly-ubiquitin chains

We report in this paper that wt ubiquitin can be detected in VICE domains. On the other hand, HA-tagged ubiquitin mutants that can only polymerize using one of the possible 7 lysines are not detected in VICE domains. This result suggests that polyubiquitinated substrates in VICE domains are not conjugated via homotypic ubiquitin linkages (Figure 6). Thus it appears that polyubiquitination of substrates in VICE domains may involve multiple ubiquitin lysine linkages that would result in the formation of branched ubiquitin chains. Interestingly, HSV-1 encodes a RING finger E3 ubiquitin ligase (ICP0) that transiently associates with VICE domains and appears to play a role in the reorganization of Hsc70 (Livingston and Weller, unpublished data [18]). Polyubiquitin chain formation mediated by ICP0 has not been described in detail. An intriguing possibility is that ICP0 may mediate the conjugation of unique branched ubiquitin chains on substrates localized to VICE domains. Specific branched polyubiquitin chains have been reported to determine the fate of the target protein via recognition by ubiquitin-binding proteins.

### Nuclear protein quality control in the absence of infection

The Gardner laboratory has described a nuclear PQC system in yeast consisting of a nuclear ubiquitin ligase (San1p) that targets four mutant misfolded proteins, but not their native counterparts, for proteolytic degradation [60]. Until recently, a mammalian homologue of San1p had not been identified. In a study by the Tsuji laboratory, the mammalian nuclear protein UHRF-2 (ubiquitin plant homeodomain RING fingers) and the yeast San1p have now been shown to promote the clearance of nuclear polyglutamine aggregates in mammalian cells and primary neurons [61]. It will be interesting to determine whether these proteins are involved in nuclear PQC in the context of HSV-1 infection.

### The ability to tolerate misfolded proteins may vary with cell type

It has been observed that some HSV-1 mutants are growth restricted in some cell types but not others. It is possible that the ability to tolerate misfolded proteins by some cells may provide the basis for this pattern of permissivity. For instance the immediate-early protein ICP22 is dispensable for productive infection in Vero cells; however, it is required for efficient infection of HEL fibroblasts [62]. Bastian et al have now demonstrated that ICP22 is absolutely required for VICE domain formation even at a high multiplicity of infection (Bastian, Livingston, Weller and Rice, unpublished results). We suggest that the requirement for VICE domain formation may vary with cell type depending on the protein processing capability of the cell. In a cell line that can efficiently process large amounts of protein, the requirement for VICE domain formation may not be as stringent as in other cells. Thus, VICE domain formation may be a mechanism by which HSV-1 can adapt to different cell types to mediate productive infection.

Another alphaherpesvirus, VZV, does not reorganize Hsc70 into VICE domains, and has thus apparently evolved a different strategy to mediate protein quality control [63]. The life cycle of VZV is much longer than that of HSV-1, and viral spreading is less efficient [64]. Because it is very difficult to obtain high titer stocks of this virus, it is possible that VZV infection is comparable to an HSV-1 infection at low MOI in that the cellular processing machinery is more capable of handling viral protein production. Under these conditions, the signal for reorganization of Hsc70 and the PQC machinery may not be triggered.

### Summary

In this paper we have demonstrated that nuclear PQC machinery is reorganized during infection by HSV-1. We and others have previously shown that functional Hsc70 is required for RC formation and efficient virus production, and formation of VICE domains occurs during the earliest stages of infection (Livingston and Weller unpublished data [20],[21]). Taken together these data indicate that HSV-1 has evolved a mechanism to commandeer the host PQC machinery using a strategy that may accomplish several goals. In addition to their potential role in sequestering dangerous signals to prevent apoptosis, VICE domains may also serve to sequester remodeling machinery that can be recruited into RC not only for the folding and assembly of viral proteins but also to cope with more extreme situations in the case of heat shock or other forms of stress. Based on these results we propose that VICE domain formation is important to promote viral infection especially under situations in which the PQC is overwhelmed.

## Materials and Methods

### Cell culture

Vero (African Green Monkey kidney fibroblast) cells were obtained from the American Type Culture Collection (ATCC) and maintained at 37°C in Dulbecco's Modified Eagle Medium (DMEM) (Invitrogen, Carlsbad CA) supplemented with 5% fetal bovine serum (FBS, Gemini Bio-Products, Woodland CA) and 1% penicillin/streptomycin (Invitrogen). For FRAP analysis, Vero cells were maintained in phenol red-free DMEM (Invitrogen). Limited passage primary human foreskin fibroblast (HFF-1) cells were purchased from the ATCC and were cultured in DMEM supplemented with 10% FBS, 1× sodium pyruvate (Invitrogen) and 1% penicillin/streptomycin (Invitrogen) at 37°C. HFF-1 cells were cultured and used for experiments prior to reaching passage 20.

### Viruses and viral infections

Strain KOS served as wt HSV-1. The phosphonoacetic acid (PAA)-resistant virus, PAAr5, was provided by Dr. Donald Coen (Harvard Medical School, Cambridge MA) [65]. The UL30 mutant virus, Hp66 [66], in which LacZ was inserted into the UL30 gene, was provided by Dr. Charles Hwang (Upstate Medical University, Syracuse NY). The ICP8-mutant virus, HD2, was provided by Dr. David Knipe (Harvard Medical School, Cambridge MA) [67]. The UL52-mutant Hr114, UL5 mutant Hr99 and UL9-mutant Hr94 viruses were previously described [68],[69],[70]. Viral infections in the presence and absence of 400 ug/mL PAA (Sigma Chemical Company) were previously described [21]. For heat shock of uninfected and infected cells, each dish of cells was transferred from 37°C to a 39.5°C or 42°C incubator for 1 hour and either transferred back to 37°C for the remainder of infection or harvested immediately for confocal immunofluorescence (IF) or Western analysis.

### Plasmids and transfection of plasmid DNA

The GFP170* expression vector was provided by Dr. Elizabeth Sztul (University of Alabama at Birmingham, Birmingham AL) [35]. The wt Hsc70-GFP expression vector [71] was provided by Dr. Hidde Ploegh (MIT, Cambridge MA). The following plasmids were purchased from Addgene (Cambridge, MA): pRK5-HA wt (plasmid 17608), K0 (plasmid 17603), K33 (plasmid 17607), K48 (plasmid 17605), K48R (plasmid 17604) and K63 (plasmid 17606) [39]. Plasmids pRK5-HA K6, K11, K27 and K29 were generated using K0 (lysines 6, 11, 27, 29, 33, 48, 63 converted to arginine) as a blank template for mutagenesis of arginine back to lysine at positions 6, 11, 27 and 29 using the Stratagene QuikChange XL Site-Directed Mutagenesis Kit. Primer sequences were as follows: R6K forward 5′-CCATGCAGATCTTCGTCAAAACGTTAACCGGTAGAAC-3′; R6K reverse 5′-GTTCTACCGGTTAACGTTTTGACGAAGATCTGCATGG-3′; R11K forward 5′-CGTCAGAACGTTAACCGGTAAAACCATAACTCTAGAAGT-3′; R11K reverse 5′-ACTTCTAGAGTTATGGTTTTACCGGTTAACGTTCTGACG-3′; R27K forward 5′-CCATCCGATACCATCGAAAACGTTAAAGCTAGAATTCAAGAC-3′; R27K reverse 5′-GTCTTGAATTCTAGCTTTAAGTTTTCGATGGTATCGGATGG-3′; R29K forward 5′-CATCGAAAACGTTAGAGCTAAAATTCAAGACAGAGAAGGCA-3′; R29K reverse 5′-TGCCTTCTCTGTCTTGAATTTTAGCTCTAACGTTTTCGATG-3′. Mutations were confirmed by sequencing analysis.

Vero cells were transfected with the indicated amount of plasmid DNA using the Lipofectamine/Plus Reagent method (Invitrogen) as recommended by the manufacturer. Following 16–18 hours of protein expression, transfected cells were either prepared for IF as described below or superinfected for an additional 6 hours before fixation.

### Immunofluorescence (IF) analysis

Preparation of cells cultured on glass slides for IF analysis was previously described [21]. Monoclonal rat-anti-Hsc70 (Stressgen SPA-815), monoclonal mouse-ant-ICP4 (Abcam ab6514), and monoclonal mouse-anti-ICP0 (East Coast Biotech H1A027) were each diluted 1∶200. Polyclonal rabbit-anti-ICP8 367 was a gift from Dr. William Ruyechan (University at Buffalo, Buffalo NY) [72] and was diluted 1∶400. Monoclonal mouse-anti-HA tag (Santa Cruz Biotechology sc7392) was diluted 1∶100.

Imaging was performed using a 63× planapochromat objective lens (numerical aperture 1.4) and a Zeiss LSM 510 Meta confocal microscope equipped with argon and helium lasers. The data shown in Figures 4 and 5 were imaged using a Zeiss LSM 410 confocal microscope and a Zeiss LSM 510 Confocor microscope, respectively. Images were arranged using Zeiss LSM 510 Meta software, Adobe Photoshop 7.0 and Adobe Illustrator 10.

### In situ detergent extraction to remove non matrix-bound proteins

Vero cells attached to glass coverslips were washed in 1× PBS and then treated for 2 min with cytoskeletal extraction buffer containing 0.5% Triton X-100 on ice followed by another PBS washing step. Cytoskeletal extraction buffer was described previously [73]. In parallel, cells were treated with PBS instead of cytoskeletal buffer as a control. Cells were fixed in 4% paraformaldehyde at room temperature for 10 min and prepared for confocal microscopy as described above.

### 20S catalytic activity reagents and microinjection

The fluorescent self-quenching protease substrate DQ-OVA (ovalbumin labeled with BODIPY FL dye) was purchased from Molecular Probes (D12053, Invitrogen, Carlsbad CA). Dextran (MW 70 kDa) conjugated to the Texas-red dye was purchased from Invitrogen. Lactacystin was purchased from Sigma Chemical Company (St. Louis, MO).

HFF-1 cells were plated in a 35 mm glass coverslip-bottom dish purchased from MatTek (Ashland, MA). HFF-1 cells were chosen for this experiment because microinjection of Vero cells resulted in clogging of the microinjection needle. No differences in VICE domain formation between HFF-1 and Vero cells have been observed. Before microinjection, serum-containing media was changed to serum-free DMEM to prevent clogging of the needle. DQ-OVA was diluted in 1× PBS to 0.25 mg/mL, a concentration tolerated by uninfected HFF-1 cells over the course of an hour (data not shown). HFF-1 cells were then co-microinjected with DQ-OVA and 70 kDa Texas Red Dextran (injection control). DQ-OVA was predigested by diluting DQ-OVA in 0.5% trypsin and incubating at 37 degrees for 1–2 hours followed by inactivation of trypsin at 65 degrees C for 20 min. To inhibit 20S proteasomal activity, HFF-1 cells were co-injected with 0.25 mg/mL DQ-OVA, TX Red Dextran (70 kDa) and 15 uM lactacystin. For microinjection of infected cells, HFF-1 cells were co-injected with 0.25 mg/mL DQ-OVA and TX Red Dextran following 5 hours of KOS infection at an MOI of 2. Microinjected cells were incubated for 20–30 min at RT and/or 37 degrees to allow cleavage of the DQ-OVA substrate before fixation and preparation for IF as described above. Hsc70 was detected using rat-anti-Hsc70 (SPA-815, Stressgen 1∶200) and Alexafluor goat-anti-rat 647 (Molecular Probes 1∶200). Imaging was performed on a LSM 510 Confocor microscope. No overlap in fluorescence detection between red, green and far-red channels was observed in control experiments.

### Western blotting of infected cell lysates

Infected Vero cells in 60 mm dishes were lysed in 2× SDS sample buffer containing DTT at a final concentration of 33 mM. Samples were boiled for 5 min and each sample was loaded to a 10% SDS-PAGE gel. Proteins were transferred to Millipore PVDF membranes followed by blocking in 5% milk/TBST. Membranes were blotted with mouse-anti-ICP4 (Abcam 1∶1000 ab6514), mouse-anti-ICP8 (Abcam 1∶1000 ab20194), mouse-anti-gC (Abcam 1∶1000 ab6509), rat-anti-Hsc70 (Stressgen 1∶10,000 SPA-815) and mouse-anti-γ tubulin (Sigma 1∶10,000 T5326) for 1 hour. Following extensive washing in 1× TBST, membranes were treated with HRP-conjugated sheep-anti-mouse (Amersham Biosciences 1∶20,000) or HRP-conjugated goat-anti-rat antibody (Zymed 1∶20,000) for 30 min. Blots were developed using Amersham Plus ECL detection reagents.

### Analysis of particle-to-pfu ratio

The titer of each viral stock indicated in Figure 2A was normalized to 1×10^∧^7 pfu/mL by dilution in 1×PBS. Viral particles were pelleted at 10,000 rpm for 40 min followed by resuspension in 1 mL 1×PBS and microfuged again 10,000 rpm for 40 min. Pellets were resuspended in 60 ul 1×PBS followed by addition of DTT-containing SDS sample buffer to a final concentration of 2×. Samples were boiled for 5 min, resolved on 8% SDS-PAGE gels, transferred to Millipore PVDF membranes and reacted with mouse-anti-VP5 (courtesy of Dr. Jay Brown) diluted 1∶1000 for 1 hour. Membranes were incubated with HRP-conjugated sheep-anti-mouse antibody (Amersham Biosciences) for 40 min and chemiluminescent signal was detected using Amersham Biosciences ECL developing solutions.

### FRAP analysis

Vero cells attached to a coverslip-embedded 35 mm dish were transiently transfected to express wt Hsc70-GFP (courtesy of Dr. Hidde Ploegh, MIT) for 16–18 hours prior to infection with KOS at an MOI of 2. At 5–6 hours post infection, media was changed to phenol red-free DMEM and infected cells were imaged using a Zeiss LSM 510 Meta confocal microscope. Total fluorescence Hsc70-GFP in VICE domains was bleached using the argon 488 nm laser at 100% laser power for 60 iterations in widefield. 120 frames were collected over the course of 190 sec at 2.5× zoom using a planapochromat 63× objective (numerical aperature 1.4) with the pinhole at maximum width to collect fluorescence from the entire depth of the specimen. Data were analyzed using Metamorph imaging software. Average fluorescence intensity in regions of interest of equal size that contained photobleached VICE domains, unbleached VICE domains, and nucleoplasm and average cell-free background was subtracted. Values were normalized to the average of 5 prebleach images, and corrected for bleaching during monitoring by normalizing to average intensity of the entire cell. Images were arranged using Adobe Photoshop and Adobe Illustrator; images shown in Figure 7 were adjusted for display by histogram stretching using identical settings for all images.

### Plaque assay

Vero cells were infected with KOS at a MOI of 10. Cells and media were collected at 12 hours post infection and frozen overnight at −70°C. Samples were freeze-thawed for a total of 3 times and clarified by centrifugation at 1500 rpm for 10 min. Supernatants were diluted serially, adsorbed to Vero cells for 1 hour and infected cells were overlayed with methylcellulose containing bicarbonate and penicillin/streptomycin until fixation in formaldehyde (4% final concentration). Methylcellulose was washed from the cell monolayer and cells were stained with 0.2% crystal violet.
